# Regulation of the Autonomic Nervous System on Intestine

**DOI:** 10.3389/fphys.2021.700129

**Published:** 2021-07-14

**Authors:** Hongyi Duan, Xueqin Cai, Yingying Luan, Shuo Yang, Juan Yang, Hui Dong, Huihong Zeng, Lijian Shao

**Affiliations:** ^1^Medical College of Nanchang University, Nanchang, China; ^2^Jiangxi Provincial Key Laboratory of Preventive Medicine, Nanchang University, Nanchang, China; ^3^Jiangxi Provincial Key Laboratory of Interdisciplinary Science, Nanchang University, Nanchang, China

**Keywords:** intestine, autonomic nerve system, enteric nervous system, cellular proliferation, intestinal disease

## Abstract

Intestine is composed of various types of cells including absorptive epithelial cells, goblet cells, endocrine cells, Paneth cells, immunological cells, and so on, which play digestion, absorption, neuroendocrine, immunological function. Intestine is innervated with extrinsic autonomic nerves and intrinsic enteric nerves. The neurotransmitters and counterpart receptors are widely distributed in the different intestinal cells. Intestinal autonomic nerve system includes sympathetic and parasympathetic nervous systems, which regulate cellular proliferation and function in intestine under physiological and pathophysiological conditions. Presently, distribution and functional characteristics of autonomic nervous system in intestine were reviewed. How autonomic nervous system regulates intestinal cell proliferation was discussed. Function of autonomic nervous system on intestinal diseases was extensively reviewed. It might be helpful to properly manipulate autonomic nervous system during treating different intestinal diseases.

## Introduction

**FIGURE 1:**
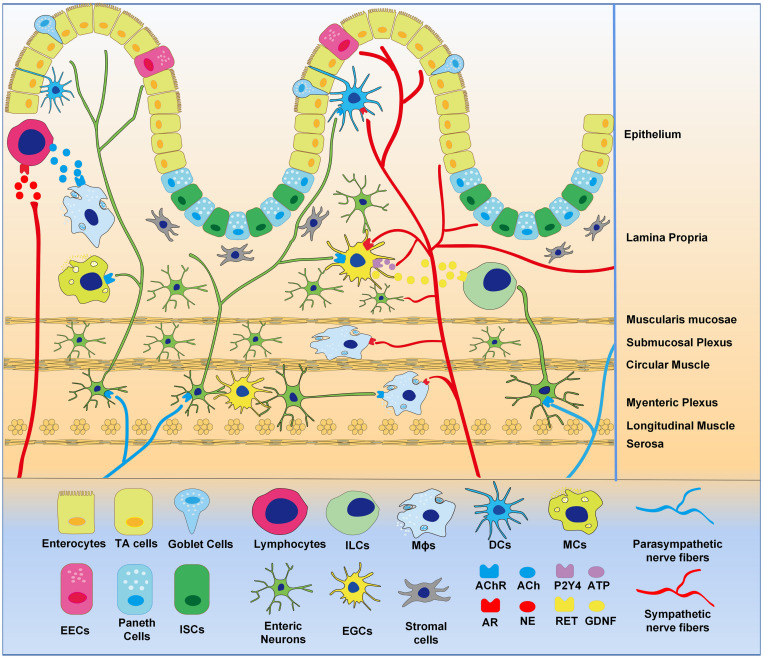
Distribution of intestinal neural network. Extrinsic sympathetic and parasympathetic nerve fibers enter the whole layer of the intestinal wall to form complex neural networks with intrinsic enteric nervous and EGCs that innervate gastrointestinal epithelial and immune cells. Epitheliums are differentiated from intestinal stem cells, demonstrating enterocytes, endocrine cells, goblet cells, and Paneth cells. Intestinal stromal cells and various immune cells reside in the intestinal wall, the later including macrophages (Mϕs), lymphocytes, mast cells (MCs), dendritic cells (DCs), and innate lymphoid cells (ILCs). Intestinal neural networks regulate intestinal cells, via neurotransmitters (ACh, NE, ATP etc.), neurotrophic factors (GDNF etc.) and receptors (AChR, AR, P2Y4, RET etc.).

Innervation of the gastrointestinal tract includes extrinsic autonomic nerve system (ANS) and intrinsic enteric nervous system (ENS). The related neurotransmitter receptors are widely distributed in various intestinal cells. The intestinal sympathetic nerve originates from the anterior vertebral ganglia and innervates the different segments of the intestine (Figure 1). The property benefits to balance various homeostasis functions, such as controlling vascular tension, regulating the activity of the smooth muscle in the intestine, reducing the secretion of the mucosa (Browning and Travagli, 2014). Intestinal vagus regulates motility in the small intestine and colon, and controls gastric motility, and secretion (Costa et al., 2000; Bohorquez et al., 2015). The regulation of gastrointestinal sympathetic and parasympathetic pathways plays crucial roles on intestinal mucosal regeneration and the immune response. Sympathetic and parasympathetic fibers entering the intestinal wall form synaptic connections with the myenteric ganglia, smooth muscle, and mucosa (Costa et al., 2000; Bohorquez et al., 2015). Sympathetic nervous system (SNS) and parasympathetic nervous system (PNS) can function independently on the gut. Nerve endings from SNS are concentrated densely at the base of the crypt where intestinal stem cells (ISCs) are located (Norberg, 1964; Gabella and Costa, 1968), while PNS nerves and intestinal epithelial cells (IECs) are directly synapsed (Bohorquez et al., 2015). The effects of norepinephrine NE on enteric epithelial cells can be transduced by SNS signaling. It is possible that SNS and PNS directly control IECs. Although both PNS and ENS use acetylcholine (Ach) as a neurotransmitter, PNS denervation with intact ENS circuits negatively affects intestinal epithelial proliferation (Mcconalogue and Furness, 1994) and inflammation (Ghia et al., 2006, 2007). These data suggest that PNS can regulate intestinal mucosal regeneration and inflammation.

The autonomic nervous system can function through intestinal stromal cells and immune cells, which include macrophages (Mϕs), lymphocytes, mast cells (MCs), dendritic cells (DCs), and innate lymphoid cells (ILCs) in intestine. They either form synapses with autonomic nerves or express the neurotransmitter receptors, which have positive or negative effects on the physiological and pathological states of the gut (Ghia et al., 2006, 2007). It has been documented that the autonomic nervous system directly or indirectly regulates the physiological activity of IEC, stromal cells, enteric nerves, and intestinal immune cells. In the present review, we will discuss the regulation of autonomic nervous system on intestine under physiological and pathological settings, which will provide new strategies to treat various intestinal diseases.

## Autonomic Nervous System Regulates Intestinal Epithelial Cell Proliferation

To maintain appropriate tissue function, IECs are rapidly renewed, a process driven by ISCs that proliferate in crypts (Barker et al., 2007). Stem cells at the bottom of the crypt divide to produce transit-amplifying (TA) progenitor cells. Once they reach the base of the villi, these cells complete differentiation and perform their maturation. Ample evidence has shown that ANS is involved in the proliferation of epithelial cells and affects intestinal homeostasis.

### Effects of Autonomic Neurotransmitters and Receptors on the Intestinal Epithelial Cell Proliferation

Sympathetic nervous system and PNS nerve endings are adjacent to or directly synapse with IECs (Gabella and Costa, 1968; Bohorquez et al., 2015). It has demonstrated that IECs express multiple autonomic neurotransmitter receptors [M1-5 (Greig and Cowles, 2017), α-2A (Paris et al., 1990; Valet et al., 1993; Schaak et al., 2000), β-2 (Zeng et al., 2020), Table 1], which are involved in the proliferation regulation of intestinal epithelium. Researchers reported that cholinergic neurons directly control the ISCs (Lundgren et al., 2011). Greig and Cowles (2017) found that the depth of ileal crypts was increased in all five knockout mice lacking muscarinic receptor subtypes (M1KO-M5KO) than that in wild-type mice. The ileal crypt proliferation index was significantly increased in four knockout mice except for M4KO mice. Schaak et al. (2000) confirmed that upregulation of α2A epinephrine receptor increased proliferation of intestinal CaCo2 epithelial cells. It is noteworthy that SNS-associated α2A receptor and PNS-associated ACh receptor subtypes M1 and M3 are elevated in ISCs when compared to those in Paneth cells (Davis et al., 2018). M3 and M5 muscarinic receptors exist on the ISCs (Lundgren et al., 2011). They may be important targets for subsequent effects on the proliferation and differentiation of ISCs.

**TABLE 1:** ANS regulates intestinal cell proliferation.

**Populations**	**Conditions**	**Autonomic nerve receptors**	**Functions**	**References**
Intestinal epithelial cells (IECs)	M1KO-M5KO	Knockout M1-5 muscarinic receptors	Increase depth of ileal crypts, increase proliferation	Greig and Cowles (2017)
	Cholinergic agonist carbachol	Activate mAChR	Reduce proliferation	Takahashi et al. (2014)
	CaCo2 cell transfected with α2A epinephrine receptor	Activate α2A adrenergic receptor	Increase proliferation	Schaak et al. (2000)
	α2 adrenergic agonist clonidine	Activate α2 adrenergic receptor	Increase proliferation	Kennedy et al. (1983)
	α adrenergic antagonist phentolamine	Inhibit α adrenergic receptor	Reduce proliferation	Kennedy et al. (1983)
	α1 adrenergic agonist phenylephrine β adrenergic agonist isoprenaline	Inhibit α-1 and β adrenergic receptors	Inhibit proliferation	Kennedy et al. (1983)
	NE and ACH treatment	Activate adrenergic receptor	Reduce the expression of the cyclin D1	Davis et al. (2018)
Intestinal stem cells (ISCs)	Mouse small intestine	M1,3 muscarinic receptors	Specifically increase in ISCs relative to villus cells or Paneth cells	Davis et al. (2018)
	Rat small intestine	M3,5 muscarinic receptors	Expression in ISCs	Lundgren et al. (2011)
	Mouse small intestine	α2A adrenergic receptor	Specifically increase in ISCs relative to villus cells or Paneth cells	Davis et al. (2018)
	ISO treatment after chemotherapy	Activate β2 adrenergic receptor	Ameliorate the reduction in ISCs during chemotherapy	Zeng et al. (2020)

Additionally, it is well known that Paneth cells and stromal cells, as the content of highly structured niche where the ISCs reside, are the sources of Wnts and R-spondins for ISCs. Particularly, stromal cells product Wnts, R-spondin 1 and R-spondin 3 to activate Wnt pathway in ISCs, which can maintain stem cells and prevent acute bowel injury (Greicius et al., 2018; Wu et al., 2021). It has been demonstrated that nerve system regulates IECs proliferation and differentiation through Paneth cells and stromal cells. For instance, acetylcholine receptor (AChR) activation regulates the function of Paneth cells (Satoh, 1988; Satoh et al., 1992), and vagal innervation is required for the function of stromal cells (Olivier et al., 2016).

Exogenous administration of primary autonomic neurotransmitters alters intestinal cell proliferation (Table 1). Alpha-2 agonists increased epithelial proliferation in the jejunum and colon in mice and rats, while phentolamine therapy (alpha-adrenergic antagonists) reduced the numbers of cells in crypts of the jejunum in rats. In contrast, α-1 and beta-adrenergic agonists inhibit cell proliferation in intestine (Tutton, 1975; Kennedy et al., 1983). These methods do not promote or inhibit the proliferation of colon cancer cells (Tutton and Barkla, 1977). Recent studies have demonstrated that NE and ACH regulate the proliferation of intestinal organs by decreasing expression of cyclin D1 (Davis et al., 2018). ACh agonists reduce the proliferation of intestinal epithelial organs (Takahashi et al., 2014). Our group showed that β2 receptor co-localized with Olfm4^+^ ISCs in crypts. Isoproterenol treatment ameliorates the reduction in the numbers of ISCs during chemotherapy (Zeng et al., 2020). This is mediated by activating β2 adrenergic receptor on ISCs (Zeng et al., 2020). Function of sympathetic nerves has also been demonstrated using genetic approaches on stem cells, such as hematopoietic stem cells (Lucas et al., 2013) and hair follicle stem cells (Shwartz et al., 2020). These data indicate that autonomic nervous system plays an important role in intestinal mucosal regeneration under pathological conditions. Autonomic co-transmitters bind to receptors on ISCs to affect cellular proliferation. For example, vasoactive peptides (Khedr et al., 2018; Iwasaki et al., 2019), neuropeptides Y (Erlinge et al., 1994; Oben et al., 2003) and adenosine triphosphate (ATP) (Erlinge et al., 1993, 1994) can regulate intestinal cell proliferation. These studies support ANS can regulate ISC proliferation and tissue regeneration through neurotransmitters and their receptors.

### Effects of Autonomic Nerve Denervation on the Proliferation of Intestinal Epithelial Cells

Surgical or chemical ablation of the branches of ANS, the sympathetic or parasympathetic nerves, causes the loss of autonomic neurotransmitter sources and alters the proliferation of intestinal crypt cells (Tutton and Helme, 1974; Musso et al., 1975; Tsibulevskii and Orlova, 1976; Lachat and Goncalves, 1978; Kennedy et al., 1983; Callaghan, 1991). The direction of proliferation changes is based on the time after denervation (Davis et al., 2017). After SNS or PNS denervation, proliferation was shown to increase (Tsibulevskii and Orlova, 1976; Lachat and Goncalves, 1978; Callaghan, 1991) and decrease (Tutton and Helme, 1974; Musso et al., 1975; Lachat and Goncalves, 1978; Kennedy et al., 1983; Callaghan, 1991) at different time points. Several studies have shown that after the removal of SNS or PNS, the gut returns to normal proliferative levels (Musso et al., 1975; Lachat and Goncalves, 1978; Davis et al., 2017), which is satisfactory information for patients who need autonomic nerve denervation. Intestinal epithelial proliferation was reduced during first 3 days in rat intestines after PNS denervation (Musso et al., 1975; Lachat and Goncalves, 1978), while the cellular proliferation in jejunum (Tsibulevskii and Orlova, 1976; Callaghan, 1991) and ileum (Callaghan, 1991) was back to normal after 7 days under PNS denervation. Previous studies have detected indirect effects on proliferation due to changes in food intake, inflammation or other factors that alter the proliferation of IECs following denervation surgery (Dailey, 2014; Slater et al., 2017; Goldstein et al., 2021). For instance, neurons expressing agouti-related protein (AgRP) are a highly active group of hypothalamuses during hunger that significantly promote eating behavior, and direct injection of nutrients into the gut leads to a sustained and rapid reduction in AgRP neuron activity (Aponte et al., 2011). Subdiaphragmatic vagotomies reducated the effect of fat on AgRP neurons activity, changing the certain food intake (Goldstein et al., 2021).

The effects of SNS denervation on intestinal epithelial proliferation seem to be related to the degree of denervation induced by different denervation techniques. Chemical denervation (6-OHDA) of sympathetic nerves resulted in a long-term reduction in proliferation (Tutton and Helme, 1974; Kennedy et al., 1983), along with a reduction in cryptic ISCs (Zeng et al., 2020). However, more precise denervation has a short-term effect in the jejunum (Tutton and Helme, 1974). Therefore, the intestinal autonomic nervous system can directly affect the proliferation of IECs at the early phase after denervation.

## Immune Regulation of Intestine by ANS System

### Intestinal Epithelial Cells (IECs)

The intestinal physical barrier was formed by IECs and the tight junction between IECs, which is the largest exchange surface between the body and the external environment. Once the intestinal epithelial barrier function is disrupted or maladjusted, it will cause bacterial translocation, leading to a severe blow to the body’s multiple organs and systems with sepsis and even death (Magnotti and Deitch, 2005). Autonomic nerve can change the barrier function in physiological and pathological states. Extradural intrathoracic lidocaine can block sympathetic nerves, inhibiting the activation of intestinal macrophages and the destruction of the intestinal mucosal barrier in endotoxemia mice (Schaper et al., 2013). Vagus nerve stimulation (VNS) reduces intestinal permeability in lipopolysaccharides (LPS)-induced endotoxemia mice and counteracted tight junction damage (Zhou et al., 2013).

The effects of parasympathetic neurotransmitters and receptors on barrier function have been demonstrated. Acetylcholine (ACh) mediates the increased colonic permeability in stress-induced barrier dysfunction in rats, while muscarinic receptor antagonists blocked the increased colonic permeability in the stressed conditions (Gareau et al., 2007). M3 receptor activation increases macromolecule transportation in mouse and human IECs (Cameron and Perdue, 2007). However, the protective effect of muscarinic receptors on the maintenance of the intestinal epithelial barrier was observed under different conditions. It was demonstrated that activation of muscarinic receptors in the monolayer of CaCO-2 epithelial cells counteracts interleukin (IL)-1β-induced barrier disruption by reducing myosin light-chain kinase protein translation (Schutz et al., 2015). Activation of muscarinic receptor with bethanechol causes the cytokine exposure of epithelial cells and a decrease in occludin expression (Slater et al., 2017). Similarly, activation of M1 or M3 muscarinic receptors counteracts the destruction of the single layer of intestinal epithelial barrier induced by inflammatory mediators (Khan et al., 2014; Uwada et al., 2017). Besides, nicotine receptors affect the maintenance of the integrity of the intestinal epithelial barrier, particularly the α7 nicotinic acetylcholine receptors (α7-nAchRs). The receptor has been shown to counteract changes in rat tight junction protein and increase intestinal permeability induced by LPS (Zhang and Li, 2012; Zhou et al., 2013). Similar findings have been observed in models of impaired barrier integrity induced by burns, in which nicotine injections prevent the reduction of burn-induced occludin and zonula occludens (ZO)-1 to maintain intestinal barrier function (Costantini et al., 2012). AChR activation mediated the function of Paneth cells (Satoh, 1988; Satoh et al., 1992), goblet cells (Gustafsson et al., 2012; Birchenough et al., 2016), and enteroendocrine cell (Anini and Brubaker, 2003), such as degranulation and secretion. AChR activation results in increased antigen presentation of goblet cells (Mcdole et al., 2012). Therefore, parasympathetic nerve plays an irreplaceable role in the regulation of intestinal barrier.

### Enteric Nervous System (ENS)

Enteric nervous system has been called “the second brain,” which be allowed to regulate intestinal behavior without central nervous system input. Although the ENS can function independently, autonomic nerve fibers entering the intestinal wall can form synaptic connections with part of the intestinal ganglion cells (Costa et al., 2000; Figure 1). Extrinsic connectivity from the CNS to the ENS is composed of sympathetic and parasympathetic nerve fibers. The ENS can regulate intestinal immunity through the synapses with enteric neuron.

Enteroglial cells (EGCs), as support cells of intestinal neurons, form a communication network in the intestinal wall via secreting neuron-related factors, regulating intestinal immunity and epithelial cell proliferation (Ochoa-Cortes et al., 2016). Researchers have recognized the role of autonomic innervation in EGCs (Figure 1). Gulbransen and Sharkey (2009) and Gulbransen et al. (2010) demonstrated that sympathetic pathways were involved in the regulation of EGCs. Sympathetic fibers activate EGCs via releasing ATP through purinergic P2Y4 receptors on EGCs. Nasser et al. (2006) found that EGCs express alpha 2 adrenergic receptor despite the functional significance of the receptor has not been assessed. The activation of EGCs attenuates the interruption of intestinal epithelial barrier through VNS (Costantini et al., 2010). EGCs secret nitric oxide, which takes part in the maintenance of intestinal homeostasis and the regulation of ion transportation in epithelial cells (Maceachern et al., 2011).

### Stromal Cells

Intestinal stromal cells, as part of stem cell niche, are also called intestinal myofibroblasts. Its important role in the IECs proliferation and differentiation has been described above. It has been shown that stromal cells express a broad range of neurotransmitter receptors in a variety of different tissues and have the ability to respond to various neurotransmitters. Thus, it is plausible that ANS innervation of the gut may be able to directly or indirectly affect intestinal stromal cells (Wheatley et al., 1992; Obara et al., 2000; Pereira et al., 2003; Méndez-Ferrer et al., 2010; Figure 1). It has been shown that colonic stromal cells were activated during acute inflammation induced by dextran sulphate sodium (DSS). Upregulation of chemokine CXCL13 and CCL20 expression in stromal cells was blocked when the extrinsic vagal innervation was blocked, implying a sense and response function of the vagus nerve (Olivier et al., 2016). Furthermore, transplantation of mesenchymal stem cells (MSCs), a hot research area, can promote *de novo* functional enteric nerve regeneration via glial cell-derived neurotrophic factor (GDNF), but not through direct *trans*-differentiation (Lin et al., 2015). These studies have revealed the possibility of bidirectional regulation of stromal cells and ANS.

### Macrophages (Mϕs)

Macrophages in the gastrointestinal tract are a highly heterogeneous population and able to sense and adapt to environmental signals (Lavin et al., 2014; Okabe and Medzhitov, 2014). Morphological studies have shown that both external autonomic nerves and intrinsic neural pathways are synaptic with muscular Mϕs (Ghia et al., 2007; Phillips and Powley, 2012; Figure 1). Cailotto et al. (2014) provided evidence that vagal efferent fibers are in contact with cholinergic neurons in the myenteric layer, while myenteric cholinergic neurons have nerve endings that are close to resident Mϕs expressing α7-nAChR. The α7-nAChR agonist, 5-hydroxytryptamine 4 receptor (5-HT4R) agonist and VNS activate the α7-nAChR receptor on Mϕs to improve gastric emptying and intestinal inflammation (de Jonge et al., 2005; The et al., 2007; Tsuchida et al., 2011). The vagal efferent fibers preferentially interact with nNOS, VIP and ChAT enteric neurons within the gut muscular rather than Mϕs. These nNOS, VIP and ChAT positive neuronal fibers are closely contacted with Mϕs (Cailotto et al., 2014), which can modulate intestinal Mϕs to produce anti-inflammatory effects, such as promoting the production of IL-10 and down-regulating the expression of iNOS in macrophages (Delgado et al., 1999). Vagus nerve activity augments intestinal macrophage phagocytosis while inhibiting immune reactivity via α4β2nAChR (van der Zanden et al., 2009). Extrinsic sympathetic neurons innervating the gut muscularis activate the β2 adrenergic receptors on muscularis macrophages, enhancing tissue-protective programs upon luminal bacterial infection (Gabanyi et al., 2016). Therefore, Mϕs are regulated by a complex set of neurons, including indirect vagal-mediated regulation, and direct regulation by the sympathetic pathway (Matteoli et al., 2014), which help to limit excessive tissue damage and inflammatory response. Therefore, Mϕs participate in an anti-inflammatory pathway mediated by sympathetic and parasympathetic pathways.

### Lymphocytes

Lymphocytes are the primary executor of the immune system in the body. Recent findings indicate that the PNS regulates the balance between different types of lymphocytes in the intestine (Figure 1). Morishita et al. (2015) demonstrated that VNS in the trauma/hemorrhagic shock model increased the Treg/Th37 ratio in the mesenteric lymph nodes after damage, which promoted tolerance to inflammation. A suppressive effect of nicotine on B-cell activation has been reported, which is mediated by α2, α4, and β2 subunits of nAChR (Skok et al., 2005). These data indicate that parasympathetic innervation protects against immune/inflammatory responses. Vagotomy inhibited the activity of Treg cells, which results in antigen tolerance impairment, accelerating the severity of colitis (Di Giovangiulio et al., 2016). The hepatic vagal afferent nerve is responsible for the indirect sensory intestinal microenvironment and transmits signal input to the brainstem solitary tract nucleus, eventually efferent to the vagus nerve and enteric neurons. The pathway maintains peripheral regulatory T cells via AChR in intestinal antigen-presenting cells (Teratani et al., 2020).

**FIGURE 2:**
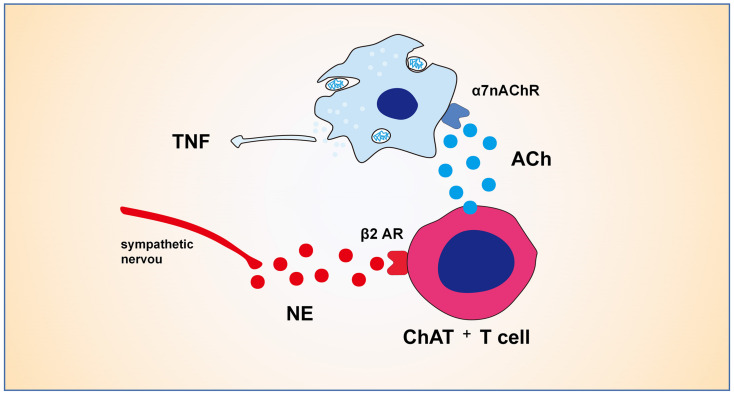
Mechanisms of cholinergic anti-inflammatory pathway. The CAIP depends on norepinephrine produced by splenic sympathetic fibers, which can lead ChAT^+^T cell to release ACh through β2 receptor. ACh from ChAT^+^T cell binds to the α7-nAChR on the bone marrow-derived non-T cells like macrophages along with inhibiting LPS-mediated cytokine production.

The adrenergic neuronal pathway has been shown to have direct and indirect effects on the activity of Treg cells. All primary and secondary lymphoid organs receive sympathetic input from post-ganglionic sympathetic fibers, such as spleen, lymphoid nodes, thymus and bone marrow (Felten et al., 1985; Nance and Burns, 1989; Bellinger et al., 1992). Activation of sympathetic nerve β2 adrenergic receptor signal impairs the differentiation and function of Th1 cells and reduces the production of IL-12, TNF-α, and IFN-γ (Sanders et al., 1997; Elenkov et al., 2000). Sympathetic fibers activate the β2 adrenaline receptor to enhance the immunosuppressive activity of Treg cells (Guereschi et al., 2013). Activation of Treg cells has been demonstrated to be mediated indirectly through DCs (Nijhuis et al., 2014). Lymphocytes play a vital role in the cholinergic anti-inflammatory pathway (CAIP) (Figure 2), which was validated by the observation that the CAIP does not occur in nude mice lacking T cells (Rosas-Ballina et al., 2011). The CAIP depends on norepinephrine produced by splenic sympathetic fibers (Rosas-Ballina et al., 2008), which can lead ChAT^+^T cell to release acetylcholine through β2 receptor (Rosas-Ballina et al., 2011; Vida et al., 2011). Acetylcholine from lymphocytes binds to the α7-nAChR on the bone marrow-derived non-T cells along with inhibiting LPS-mediated cytokine production (Olofsson et al., 2012). Therefore, intestinal sympathetic innervation may play an important role in Treg cells by activating the β2 adrenal receptor to maintain the intestinal tolerance response mediated by immunosuppression of Treg cells. These data suggest that both cholinergic and adrenergic pathways are involved in lymphocyte proliferation and function in the process of intestinal inflammation.

### Others

In addition to the above-mentioned cells, MCs, DCs, and ILCs could be regulated by ANS (Figure 1). MCs are a type of intestinal immune cells that control neuroimmune activity through two-way communications. Anatomically, the vagus nerve penetrates the intestinal mucosa and contacts MCs, while vagotomy reduces MCs density (Stead et al., 2006). Activation of nAChRs can inhibit IgE-induced bone-marrow-derived MC degranulation in mice (Kageyama-Yahara et al., 2008). VNS can induce an increase in histamine-immune response in intestinal mucosal MCs, which may be mediated by a decrease in MC degranulation (Stead et al., 2006). Lipid-rich enteral nutrition weakened intestinal MC activation in the post-operative ileus (POI) model and the LPS-induced acute enteritis model via stimulating vagal pathway (Lubbers et al., 2009; de Haan et al., 2013).

Intestinal DCs participate in antigen presentation, regulate lymphocyte differentiation, and simultaneously express multiple neurotransmitter receptors, allowing regulation by nerves (Alpaerts et al., 2015). For example, β-2 adrenal receptor activation stimulates the anti-inflammatory properties of DCs *in vitro*, which are evidenced by enhancing endocytosis and increasing the production of IL-10 (Nijhuis et al., 2014). The DCs express M3, M4, and M5 receptors. Cholinergic agonist Carbachol induces DC differentiation, increasing expression of HLA-DR and CD86 and enhancing TNF-α and IL-8 production, which are completely blocked by atropine (Salamone et al., 2011). Finally, ILCs are primarily associated with ENS. Neurotrophic factors or neuropeptide was demonstrated to have potentiality of immunoregulation for ILCs. EGCs secret various neurotrophic factors of the GDNF family, which induces the expression and secretion of IL-22 in ILC3 cells. This supports intestinal host defense and homeostasis via the production of mucin and antimicrobial peptides by epithelial cells. Neuroregulatory receptor RET, expressed in intestinal ILC3 cells, controls the activation of IL-22 and ERK-3, which is downstream factors of the p38MAPK/Akt signaling (Ibiza et al., 2016). ILC2 cell expresses multiple neuropeptide receptors including NMUR1. The expression of NMUR1 was increased under infection. Neuropeptide NMU plays an important role in the production of IL-25 and IL-33, and activation of ILC2-mediated immunity, such as responses to lung and intestinal helminth infections (Cardoso et al., 2017; Wallrapp et al., 2017). Collectively, ANS has a potential function to regulate the immune function of the intestinal cells.

## Parasympathetic Nerves System and Intestinal Diseases

Cholinergic anti-inflammatory pathway was first identified by Borovikova et al. (2000) Vagal nerve stimulation protects against lethal septic shock induced by LPS. Afferent vagal activity mediates a cytokine-induced pyretic response (Fleshner et al., 1998). The effects of CAIP on intestinal immune function may include: indirectly regulating intestinal inflammation via altering systemic inflammation through splenic nerve (Huston et al., 2006; Rosas-Ballina et al., 2008; Figure 2), or directly regulating intestinal inflammation through parasympathetic nerve (Matteoli et al., 2014). We will discuss the anti-inflammatory effects of PNS in various diseases below.

### Inflammatory Bowel Disease (IBDs)

Inflammatory bowel diseases (IBDs), including Crohn’s disease and ulcerative colitis, are complex diseases at the clinical, immunological, genetic, molecular and microbial levels. Parasympathetic innervation is involved in the pathophysiology process of IBDs in animal models and patients. A clinical study found vagal nerve dysfunction in 45% of tested patients with ulcerative colitis (UC) (Lindgren et al., 1993). Ghia et al. (2006) showed that subdiaphragmatic vagotomy aggravated acute experimental colitis in mice treated with DSS for 5- or 3-day-intrarectal DNBS injection. Subdiaphragmatic vagotomy increased the likelihood and severity of acute episodes in chronic experimental colitis (Ghia et al., 2007). O’Mahony et al. (2009) showed that subdiaphragmatic vagotomy aggravated acute experimental colitis after 5 days of DSS administration. The severity of colitis caused by acute DSS in α7-nAChR^–/–^ mice increased in the depression mouse model, and was relieved after treatment with choline chloride (α7-nAChR-specific agonist) (Ghia et al., 2009). However, data have demonstrated that vagotomy has no significant effect on the course of colitis in mice treated with DSS for 7 days (Willemze et al., 2018). These differences can be explained by the discrepancy in duration of treatment, methods of experimental colitis induced, and the vagotomy. Opposite of vagotomy is VNS, which has been shown to activate the CAIP and alleviate inflammatory bowel disease. Chronic VNS activates the choline anti-inflammatory pathway, with MAPK/NF-κB signaling pathway attenuating experimental colitis (Sun et al., 2013). A clinical study showed that long-term VNS improved vagal tone in 5 of the 7 patients with Crohn’s disease (Bonaz et al., 2016). In addition to VNS, whole-body nicotine therapy can inhibit acute DSS-induced colitis by down-regulating pro-inflammatory cytokine IL-6 and TNF-α (Ghia et al., 2006; Hayashi et al., 2014). Acetylcholinesterase inhibitors such as neostigmine, physostigmine, galantamine can attenuate intestinal damage in a model of DNBS-induced colitis in rats (Miceli and Jacobson, 2003; Ji et al., 2014). Lipid-rich enteral nutrition via activation of vagus nerve regulated mucosal MC activation to alleviate LPS-induced acute enteritis in mice (de Haan et al., 2013). A recent study found that ultrasound therapy can alleviate DSS-induced colitis through the α7-nAChRs-mediated CAIP (Nunes et al., 2019). These data suggest that vagal dysfunction plays an important role in the pathogenesis of IBDs. Inhibition of vagal activity by inhibitors, denervation, and gene editing may aggravate IBDs. Conversely, the CAIP can be activated by physical stimulation and receptor activators, alleviating colitis, in which α7-nAChR receptors may play an important role.

### Irri’ Bowel Syndrome (IBS)

Irri’ bowel syndrome (IBS) is one of the most common disorders of gut-brain interaction. ANS may be an effective target for the treatment of IBS. The abnormality of the autonomic nerve is an objective physiological factor of IBS (Cheng et al., 2013). The results from clinical studies demonstrated the impaired parasympathetic activity and enhanced sympathetic tone in patients (Pellissier et al., 2010; Chalaye et al., 2012; Mazur et al., 2012; Tanaka et al., 2018). Mood regulation can alter autonomic dysfunction in IBDs patients, whereas autonomic dysfunction persists regardless of mood regulation in IBS patients (Pellissier et al., 2010). Antidepressants can improve the gastrointestinal and psychological status of patients with IBS and improve the stress response of ANS (Wan and Chen, 2010). VNS is considered a potential therapeutic approach for IBS. Transcutaneous electrical vagal nerve stimulation with deep slow breathing, combined with electrical and physiological modulation of vagal tone, enhanced gastroduodenal motility and reduced somatic pain sensitivity (Frokjaer et al., 2016).

### Post-operative Ileus (POI)

Post-operative ileus, characterized by gastrointestinal motor dysfunction accompanied by nausea, impaired oral feeding, vomiting, abdominal distension and delayed expulsion of stool or flatus, is a frequent complication of abdominal surgery (Vather et al., 2014). The pathogenesis of POI mainly includes inflammatory responses in the intestinal wall and autonomic nerve disorders represented by increased sympathetic activity and impaired parasympathetic activity (Bauer and Boeckxstaens, 2004). In a mouse model of POI, neuroanatomical evidence demonstrates the existence of the vagal anti-inflammatory reflex in the intestinal wall. Abdominal surgery resulted in subtle inflammation of intestine, where sensory and motor vagal neurons are activated during POI. The vagus nerve is mainly output to the inflammatory region, and 42% of motor neurons innervating the intestine expressed c-fos in contrast to 7% of splenic nerve (Cailotto et al., 2012). Nicotine produces an anti-inflammatory effect on peritoneal macrophages by activating α7-nAChR. VNS improves inflammation and post-operative bowel obstruction in the POI model by activating STAT3 pathways in intestinal macrophages, both of which are anti-inflammatory by the α7-nAChR-mediated Jak2-Stat3 signaling pathway (de Jonge et al., 2005). VNS failed in the alpha-7nAChR^–/–^ mouse POI model (Matteoli et al., 2014), emphasizing the vagus nerve’s anti-inflammatory effects mediated by α7-nAChR. Stimulation of nicotine cholinergic receptors in the POI model reduces the intestinal inflammatory response and counteracts the delayed gastric emptying (The et al., 2007). Administration of lipid-rich enteral nutrition relieves POI by activating vagus nerve, dampening pro-inflammatory cytokine secretion (Lubbers et al., 2009). 5-HT4R agonists mediate ACh release from intestinal neurons, which reduces inflammatory activity in macrophages in POI rats (Tsuchida et al., 2011), suggesting that not only vagus nerve produces an anti-inflammatory effect, ENS also mediates the CAIP. Activation of the vagus nerve alleviates inflammation and symptoms of post-operative intestinal obstruction. Therefore, regulation of ANS might be a new clinical strategy for the prevention of post-operative intestinal obstruction.

## Sympathetic Nerves System and Intestinal Diseases

Sympathetic nervous system appears to play a more important role in intestinal inflammatory response compared with PNS. CAIP requires the involvement of the sympathetic nerves (Rosas-Ballina et al., 2008). Depletion of noradrenaline by reserpine in rats prevented the anti-inflammatory action of vagal stimulation (Rosas-Ballina et al., 2008). Patients with UC showed hypertonic sympathetic in IBDs (Maule et al., 2007), while the loss of sympathetic fibers was discovered in CD patients (Straub et al., 2008). The changes in the SNS may be related to the occurrence and outcome of IBDs. Sympathectomy (6-OHDA) alleviated acute DSS colitis and aggravated chronic DSS colitis in wild-type mice and chronic colitis in Il-10^–/–^ mice (Straub et al., 2008). Willemze et al. (2018) reported that sympathectomy rather than vagus nerve has a significant damaging effect on acute DSS-induced colitis in mice, while electrical stimulation of superior mesenteric nerve improves colitis. Distinctness in neurotransmitter concentrations due to different approaches to sympathectomy and the variable expression of adrenergic receptor subtypes in the intestinal inflammatory process explain the conflicting results. The study found that β2 and β3 adrenaline receptors could act as anti-inflammatory agents at high levels of norepinephrine (Borger et al., 1998; Vasina et al., 2008; Nijhuis et al., 2014), whereas α2 receptors had a relatively high norepinephrine affinity (Bai et al., 2009). Catecholamines have a significant effect on the composition of the microbial population in the lumen and its interaction with epithelial cells, which may affect the outcome of intestinal immunity (Lyte et al., 2011).

Sympathetic nerves may be involved in chemotherapy-induced intestinal mucositis. Chemotherapy drugs have been documented to damage the sympathetic nerve (Lucas et al., 2013). 5-FU-induced damage to the intestinal sympathetic nerve was dose-dependent and resulted in a decrease of ISCs. 6-OHDA reduced the numbers of ISCs in the mouse jejunal crypt along with a reduction of numbers of Paneth cells. Isoproterenol (ISO), activating the beta-2 adrenergic receptor on the ISCs, ameliorated the damage to the intestine after chemotherapy, which is mediated by decreasing cellular apoptosis and protecting intestinal sympathetic nerve (Zeng et al., 2020). Thus, activation of sympathetic pathways or neuroprotective drugs may be a new therapeutic strategy to prevent the side effects of chemotherapy.

## Conclusion and Future Application of Autonomic Nerve Regulation on Intestine

The regulation of intestinal homeostasis by ANS may be a valuable tool in the clinic and open a new treatment strategy for various refractory or chronic intestinal diseases. To investigate the effects of ANS on the intestine, we need to understand two major effects of ANS: (1) the regulation of IEC proliferation and (2) the immune system in intestine.

Autonomic nerve system regulates IEC proliferation and plays an effective role in intestinal mucosal regeneration after trauma, surgery and infection. ANS signaling induces stem cell proliferation and differentiation, which maintains different types of epithelial cells and affects intestinal absorption spectrum. Some cancers in the gastrointestinal tract, such as colon cancer, are thought to originate from stem cells, which maintain similar properties to ISCs under tumor conditions (Barker et al., 2009; Merlos-Suarez et al., 2011; Schepers et al., 2012; Cortina et al., 2017; Shimokawa et al., 2017). Several intestinal cancer cells express autonomic receptors, and neurotransmitters or receptor activators, which might affect tumor cell proliferation (Park and Cho, 2008; Huang et al., 2012; Coelho et al., 2015; Wang et al., 2016). ANS regulation might be used as a new target to develop an effective anti-cancer strategy. ANS can be protected and activated in the intestine through drugs, preventing the short- and long-term complications after chemotherapy. Because post-chemotherapy intestinal nerve injury may be involved in the formation of intestinal inflammation, isoproterenol has been shown to ameliorate post-chemotherapy intestinal injury (Zeng et al., 2020). Therefore, the ANS signaling can effectively reduce the intestinal damage after chemotherapy. These will increase the quality of life and survival rate of cancer patients. In conclusion, understanding the role and mechanism of ANS in regulating IECs might help to increase current therapeutic strategies and achieve breakthroughs in clinical practice.

Autonomic nerve system, as a bridge between CNS and gut, may be a better therapeutic target relative to unmanageable CNS or complex intestinal environment. Autonomic disorders, such as IBDs, IBS and POI, characterized by impaired parasympathetic activity and enhanced sympathetic tone in patients. The immune/inflammatory response of ANS to intestine has been used in clinical trials. Researchers have identified the role of CAIPs in POI and CD (Bonaz et al., 2016; Stakenborg et al., 2017). Stakenborg et al. (2017) present abdominal vagal nerve stimulation as a treatment for post-operative intestinal obstruction. Abdominal VNS attenuates LPS-induced IL-6 and IL-8 elevations in whole blood collected on day 1 after colectomy without significant side effects in colectomy patients. VNS is safe and effective for patients with post-operative intestinal obstruction and has certain anti-inflammatory effects. VNS normalizes vagal tone regardless of the initial state of activation, which is advantageous in patients with a low vagal tone.

Although there is increasing numbers of the studies about ANS regulation for intestine, the controversy exists in the complete neural circuit of ANS regulation. How do we coordinate many roles of ANS on the gut? Does CAIP work through the spleen nerve or via the vagal nerve extending into the intestinal wall? Where the SNS are located in the CAIP? Further mechanism of sympathetic and parasympathetic regulation of inflammation is needed, which will obtain efficient regulation methods, enabling ANS-mediated regulation of intestinal homeostasis.

## Author Contributions

HoD and LS designed the study. All authors drafted the manuscript, read and approved the manuscript.

## Conflict of Interest

The authors declare that the research was conducted in the absence of any commercial or financial relationships that could be construed as a potential conflict of interest.
